# Anti-Inflammatory and Anticancer Effects of Anticoagulant Therapy in Patients with Malignancy

**DOI:** 10.3390/life13091888

**Published:** 2023-09-10

**Authors:** Vincenzo Russo, Luigi Falco, Viviana Tessitore, Alfredo Mauriello, Dario Catapano, Nicola Napolitano, Moiz Tariq, Alfredo Caturano, Giovanni Ciccarelli, Antonello D’Andrea, Antonio Giordano

**Affiliations:** 1 Cardiology Unit, Department of Medical Translational Science, University of Campania “Luigi Vanvitelli”—Monaldi Hospital, 80126 Naples, NA, Italy; 2 Sbarro Institute for Cancer Research and Molecular Medicine, Center for Biotechnology, College of Science and Technology, Temple University, Philadelphia, PA 19122, USA; 3 Department of Advanced Medical and Surgical Sciences, University of Campania Luigi Vanvitelli, Piazza Luigi Miraglia 2, 80138 Naples, NA, Italyantonellodandrea@libero.it (A.D.); 4 Cardiology Unit, Umberto I Hospital, 84014 Nocera Inferiore, SA, Italy

**Keywords:** malignancy, cancer, thrombosis, anticoagulants, inflammation, low-molecular-weight heparins, direct oral anticoagulants

## Abstract

Optimizing the anticoagulation therapy is of pivotal importance in patients with a malignant tumor, as venous thromboembolism (VTE) has become the second-leading cause of death in this population. Cancer can highly increase the risk of thrombosis and bleeding. Consequently, the management of cancer-associated VTE is complex. In recent years, translational research has intensified, and several studies have highlighted the role of inflammatory cytokines in cancer growth and progression. Simultaneously, the pleiotropic effects of anticoagulants currently recommended for VTE have emerged. In this review, we describe the anti-inflammatory and anticancer effects of both direct oral anticoagulants (DOACs) and low-molecular-weight heparins (LWMHs).

## 1. Introduction

Cancer patients are at a high risk for venous thromboembolism (VTE). After being diagnosed with a malignant tumor, the thrombotic risk increases quickly and remains high over time [1]. The advances in cancer treatment and earlier diagnosis have contributed to a longer life expectancy, and cancer-related thromboembolic events have emerged as the second-leading cause of death [2]. Peculiar features of VTE in cancer patients can possibly explain the data concerning the morbidity and mortality. The incidence of bleeding and recurrent VTE, despite appropriate anticoagulation, is high. Additionally, VTE events are linked to more hospitalizations and can hinder the delivery of lifesaving treatments.

Traditionally, vitamin K antagonists (VKAs) were used in conjunction with rapid-acting parenteral anticoagulants such as low-molecular-weight heparins (LWMHs), fondaparinux, or unfractionated heparin (UFH) to treat acute VTE. However, this strategy presented challenges in cancer patients due to many drug–drug interactions (DDIs) that either attenuated or amplified the anticoagulant effect of VKAs, as well as variability in gastrointestinal (GI) absorption caused by vomiting, diarrhea, or malnutrition. VKAs have a long half-life, which can make therapy more challenging in the context of thrombocytopenia or surgery, common conditions among cancer patients. In the early 2000s, for the first time, a randomized controlled trial (RCT) showed the superiority of dalteparin monotherapy versus warfarin in preventing recurrent VTE in cancer patients, without increasing the bleeding risk [3]. Recently, a meta-analysis, including twenty-three RCTs with 6980 patients, established the beneficial effects of LWMH over VKAs [4]. Nevertheless, LWMHs have some limitations such as high cost, uncomfortable route of administration, and reduced adherence to a long-term treatment [5].

Direct oral anticoagulants (DOACs) have overcome the drawbacks of both VKAs and parenteral LWMHs, i.e., oral administration without the need for routine blood monitoring, good therapeutic adherence, and fewer intracranial hemorrhages. Based on this evidence, DOACs are considered the first choice when anticoagulation therapy is needed in different clinical scenarios [6,7,8,9], even in that subgroups of patients under-represented in the RCTs [10,11,12,13,14,15,16,17,18,19,20,21,22,23]. Recently, DOACs were tested in cancer patients [24,25,26,27,28], and according to the international guidelines, rivaroxaban, apixaban, and edoxaban are considered the first-line anticoagulant therapy for patients with malignancy, except for those with GI and genitourinary cancers [29,30]. Previous experimental studies suggested “pleiotropic” effects on inflammation pathway and cancer-progression of both parenteral and oral anticoagulant therapies. The present narrative review aimed to describe anti-inflammatory and anticancer effects of anticoagulant therapy in patients with malignancy.

## 2. Cancer, Inflammation, and Thrombosis

### 2.1. Role of Inflammation in Cancer

Inflammation is a protective process in vascularized tissues that acts against foreign bodies, pathogens, or injuries. It serves to rid the host of both the initial cause of cell injury, i.e., microbes or toxins, and necrotic byproducts of inflammatory response. The mediators of inflammation include phagocytic leukocytes, antibodies, complement proteins, chemokines, and cytokines. Most of these normally circulate in an inactive state in the blood, from which they can be rapidly recruited to the damaged tissue. Inflammation typically develops through five sequential steps: (1) recognition of the noxious agent by receptors on sentinel cells, such as tissue macrophages or mast cells, resulting in the activation of these cells and subsequent release of inflammatory mediators, which trigger an immune response; (2) recruitment of leukocytes and plasma proteins into the tissues; (3) removal of pathogenic noxa mainly by phagocytic cells, which ingest and destroy microbes and dead cells; (4) regulation of the response involves resolution of inflammation once it has achieved its purpose; and (5) repair of damaged tissues. Cytokines are the main signaling molecules released by inflammatory cells and work at different steps in the inflammation pathway. They are classified into proinflammatory cytokines, such as Interleukin-1 (IL-1), IL-6, IL-15, IL-17, IL-23, tumor necrosis factor alpha (TNF-α), and interferon gamma (IFN-γ), and anti-inflammatory cytokines, such as IL-4, IL-10, IL-13, and transforming growth factor beta (TGF-β). C-C motif chemokine ligand 2 (CCL2) and C-X-C motif chemokine 12 (CXCL12) are small molecules necessary for the recruitment of inflammatory cells to the inflammatory area [31]. Rudolf Virchow was the first to describe a correlation between cancer and inflammation in 1863, noting that tumors arose at sites of chronic inflammation and that inflammation caused increased cell proliferation [32]. On one hand, inflammation has a protective role against cancer by mediating the direct elimination of mutated cells and enhancing the response to anticancer therapies. On the other hand, chronic, dysregulated, persistent, and unresolved inflammation has been associated with an increased risk of malignancy, as well as the malignant progression of cancer in most types of cancer. In the inflammatory tumor microenvironment (TME), there is an increase in DNA and protein damage, activation of oncogenes, and release of ROS which ultimately affect multiple signaling pathways such as nuclear factor-kB (NF-kB), Kirsten rat sarcoma virus (K-RAS), Janus kinase/signal transducer, and activator of transcription 3 (JAK/STAT3). These activated transcription factors mediate the expression of key cytokines and chemokines, including TNF-α and IL-6, as well as other proinflammatory enzymes. Cyclooxygenase 2 (COX-2) is upregulated in several malignancies. COX-2-generated prostaglandins enhance the migration of cancer cells while limiting apoptosis. Additionally, they promote neo-angiogenesis. In TME, cancer cells can not only prevent dendritic cells from presenting tumor-associated antigens but also recruit many immunosuppressive cells. In turn, these immunosuppressive cells provide a rich proangiogenic and pro-tumoral microenvironment, preventing both innate and specific immune response. Autocrine and paracrine effects of the cytokines released within the tumor microenvironment might further sustain these cells [33,34]. Several studies have shown that up to 25% of cancers are related to chronic inflammatory diseases [35]. Plenty of chronic inflammatory diseases are considered fully fledged precancerous environments. Therefore, targeting inflammation has become extremely important. However, a thorough understanding of the underlying molecular pathways is crucial for the development of targeted therapies.

Cytokines and chemokines have a direct effect on cancer cells and contribute to the stimulation of epithelial-to-mesenchymal transition (EMT), promoting metastases. TNF-α stimulates cell growth, survival, invasion, metastasis, and neo-angiogenesis. IL-6 has both local and systemic tumorigenic actions mediated by JAK/STAT3 pathway. Janus kinases are a family of four intracellular nonreceptor tyrosine kinases (JAK1, JAK2, JAK3, and TYK2) that act as primary signal transducers from cell surface receptors activated by cytokines and growth factors. These kinases in turn phosphorylate STAT proteins on tyrosine residues, enhancing their function as transcription factors in the expression of several genes involved in cell survival.

IL-6 seems to accelerate cell proliferation by activating inflammasome via JAK2/STAT3/SRY-Box Transcription Factor 4 (Sox4)/NOD-like receptor protein 3 (NLRP3) pathways both in vitro and in vivo [36].

IL-1α is associated with a less differentiated and more aggressive cancer through the activation of vascular endothelium and infiltration by tumorigenic inflammatory cells. IL-8 promotes tumorigenesis by inducing migration of tumor cells, promoting angiogenesis, and enhancing metastasis in patients with melanoma. Chemokine receptors are highly expressed by several malignancies and facilitate the spread of cancer cells throughout the body [37].

Several studies have found that monocyte chemotactic protein-1 (MCP-1) expression in TME is associated with tumor development, tumor invasion and metastasis, angiogenesis, and immune cell infiltration. MCP-1 exerts its effects mainly via the MCP-1/CC2 axis and leads to the activation of classical signaling pathways, such as Phosphoinositide 3-kinase (PI3K)/Protein kinase B (PKB, or Akt)/Mammalian Target of Rapamycin (mTOR), Extracellular signal-regulated kinase (ERK)/Glycogen synthase kinase 3 beta (GSK-3β)/Snail, RAF proto-oncogene serine/threonine-protein kinase (c-Raf)/Mitogen-activated protein kinase kinase (MEK)/ERK, and Mitogen-activated protein kinases (MAPKs) in different cells [38]. The NF-κB transcription factor family has a major role in inflammation. It is activated by canonical and non-canonical signaling pathways, which differ in both signaling components and biological functions [39]. Activation of non-canonical pathways has been shown to support tumor progression in multiple cancers, both solid and hematological [40]. In most tumors, NF-κB is constitutively active and activates antiapoptotic and cell-cycle progression genes, such as B cell leukemia/lymphoma 2 (BCL2), and promotes the expression of the hypoxia-inducible factor-1α (HIF-1α) and favors cell migration and invasion [34].

### 2.2. Role of Inflammation in Thrombosis

Thrombosis, inflammation, and cancer are disjointed physiological processes with a deep interdependence [41]. Circulating blood platelets are crucial to each process. Recent studies suggest that thrombosis has an important role in immune defense. Hence, the concept of immunothrombosis arose [42]. The host defense system is not limited to a strong immune response but also includes thrombosis promotion. Immunothrombosis indicates an innate immune response induced by the formation of thrombi within micro-vessels. The interaction between platelets and immune cells is supported by coagulation factors and complement system and generates an intravascular network that facilitates the recognition, containment, and destruction of pathogens [43]. When a vascular injury occurs, platelets are recruited to the site of injury by a process involving the interaction of specific platelet receptors with sub-endothelial matrix proteins [44].

After this initial phase, platelets become activated and adhere tightly to the vessel wall and release intracellular granules, which promote their own activation. The continuous processes of adhesion, activation and aggregation of platelets causes rapid growth and stabilization of thrombus [45].

Simultaneously with platelet activation, the coagulation cascade is activated at the site of the damage. The active contribution of innate immune cells to clot formation is also very important.

Platelets and clotting factors activate innate immune cells. Within a developing clot, platelets and coagulation byproducts meticulously regulate the effective functions of innate immune cells.

Indeed, monocytes and neutrophils are rapidly recruited to the endothelium and are included in growing intravascular clots. Recruitment of innate immune cells proceeds throughout the thrombus development. Platelets produce a myriad of proinflammatory cytokines and chemokines. Activated platelets also express IL-1β, which induces tissue factor (TF) expression in endothelial cells and stimulates the expression of endothelial–leukocyte adhesion molecules [46]. Additionally, IL-1β further promotes platelet activation via the IL-1 receptor. Platelets adhere to von Willebrand factor (VWF) and attach to endothelial cells, and this interaction causes tethering and rolling of leukocytes onto the endothelial surface. In this way, platelets not only lead leukocytes to a site where inflammation potentially causes atherosclerosis but also contain depots of proinflammatory mediators, such as thromboxanes and CD40 ligand [47]. An equally important role is played by proinflammatory cytokines. IL-6 and IL-1 can activate the coagulation cascade and increase fibrin deposition, through TF, and interact with antithrombin III (AT III) and protein C with a downregulating effect [48]. Moreover, coagulation proteases TF, FXa, and FIIa are key modulators of the systemic inflammatory response [49], through the activation of PARs. PARs belong to the superfamily of G-protein-coupled receptors and are located on platelets, leukocytes, and endothelial cells [50]. Four isoforms of PAR (PAR1–PAR4) have been isolated. FIIa activates PAR-1, PAR-3, and PAR-4. FXa interacts with PAR-1 and PAR-2 [51,52,53]. FXa is a vitamin-K-dependent serine protease that leads to the generation of thrombin (FIIa) from prothrombin (FII) and is a common point between the intrinsic and extrinsic coagulation pathways. Recent studies have demonstrated that FXa is also a key modulator of systemic inflammatory response, through PARs activation and NF-kB transcription [54,55]. PARs activation induces the degradation of I-κB, an inhibitor protein which causes translocation of NF-kB to the nucleus, to activate promoter regions of many proinflammatory genes. FXa increases the production of IL-6, IL-8, and some chemokines, such as MCP-1 [56,57]. The expression of adhesion molecules, such as E-selectin, intercellular adhesion molecule-1 (ICAM-1), and vascular cell adhesion molecule-1 (VCAM-1) is upregulated, promoting leukocyte migration and recruitment during inflammation [58]. Finally, FXa fosters the pro-fibrotic response, increasing the release of TGF β, fibronectin, and platelet-derived growth factor (PDGF) by fibroblasts [59,60]. FIIa is a serine protease that promotes the conversion of fibrinogen to fibrin monomers, which, upon polymerization, form a fibrin clot. Some studies have revealed FIIa-mediated proinflammatory activities in vitro, such as the production of IL-6 and IL-8 by monocytes and of chemokines, i.e., platelet activating factor (PAF) and MCP-1, by platelets and monocytes [57,61].

## 3. Indications for Anticoagulant Therapy in Cancer Patients

Cancer is associated with an increased risk of both hemorrhagic and thromboembolic complications. Several factors, including patient characteristics, cancer type and location, comorbidities, adverse effects (AEs) of antineoplastic agents and drug–drug interactions (DDIs) between antineoplastic drugs and anticoagulants may influence both hemorrhagic and thromboembolic risks. Therefore, the initiation and selection of an anticoagulant regimen require careful consideration. Over the years, there have been many concerns about the use of anticoagulants in cancer patients. The recent international guidelines on cardio-oncology provide a clear path forward for different clinical conditions [62].

### 3.1. Treatment and Secondary Prevention of Venous Thromboembolism

Apixaban, edoxaban, or rivaroxaban are recommended for the treatment of incident or symptomatic VTE in cancer patients without contraindications such as unoperated gastrointestinal or genitourinary cancer, history of recent bleeding or within 7 days of major surgery, significant thrombocytopenia (platelet count < 50,000/µL), severe renal dysfunction (CrCl, 15 mL/min), or gastrointestinal complications (Class I; Level of Recommendation: A). LMWHs are recommended for the treatment of symptomatic VTE in cancer patients with platelet count > 50,000/µL (Class I; Level of Recommendation: A) [62]. Several RCTs and meta-analyses showed that VKAs are associated with a higher risk of recurrent VTE and similar risk of major bleeding compared to LMWHs [62]. The minimum anticoagulation regimen is six months, and it can be extended in the presence of active malignancy, metastatic disease, or chemotherapy use. However, due to high risk of bleeding, cancer patients should be subjected to a periodic assessment of the risk/benefit ratio. The duration of anticoagulant regimen in patients with catheter-associated thrombosis depends on whether the catheter is removed or remains in situ. If the catheter is removed, the anticoagulation regimen should continue for a minimum of three months and until the resolution of the thrombus is confirmed with cardiac imaging. If the catheter remains in situ, long-term therapeutic anticoagulation should be continued (Class I; Level of Recommendation: C) [62].

### 3.2. Primary Prevention of Venous Thromboembolism

Low-dose anticoagulation is required in patients undergoing surgery and those hospitalized or in prolonged bed rest (Class I; Level of Recommendation: B). LMWHs used as primary thromboprophylaxis for 4 weeks after a major abdominal or pelvic cancer surgery have favorable outcomes as suggested by the ENOXACAN (enoxaparin and cancer) II study (Class I; Level of Recommendation: B) [63]. No data suggest the superiority of any LMWH over the others. Ambulatory cancer patients at high risk of thrombosis receiving systemic therapy without significant contraindications may consider primary thromboprophylaxis with a direct oral anticoagulant (DOAC) (apixaban or rivaroxaban) or LMWH (Class IIb; Level of Recommendation: B). VTE risk should be individually determined using assessments such as the Khorana [64] or the COMPASS-CAT [65] score. Apixaban (2.5 mg twice a day) treatment resulted in a significantly lower rate of VTE, although the occurrence of major bleeding episodes was higher than with a placebo [66]. Rivaroxaban (10 mg once a day) therapy resulted in a non-significant lower incidence of VTE or death due to VTE with no significant differences compared to the placebo regarding bleeding risk [67].

### 3.3. Atrial Fibrillation

Cancer patients have a higher risk of developing atrial fibrillation (AF) [62]. In the general population, the choice to start anticoagulant therapy is based on risk stratification for stroke/systemic embolism through CHA2DS2-VASc score [68]. The CHA2DS2-VASc score has not been validated in cancer patients [69]. The CHA2DS2-VASc score in cancer patients should not be used to indicate the initiation of anticoagulant therapy based on the risk stratification for stroke/systemic embolism but to identify patients at low thrombotic risk who can avoid anticoagulation [70]. For bleeding risk assessment, the HAS-BLED score may be considered [62]. A proposed approach to anticoagulant therapy in cancer is based on the acronym T (thrombotic risk), B (bleeding risk), I (drug interactions), P (access and patient preferences) [71].

VKA use in cancer patients is limited by their drawbacks and remains indicated only in patients with a mechanical prosthetic valve or moderate to severe mitral stenosis (Class IIa; Level of Recommendation: B).

LMWHs represent a viable short-term anticoagulation option, in particular in hospitalized patients with a recent cancer diagnosis, advanced-stage cancer, or during cancer therapy [62]. However, no data suggest LMWHs’ efficacy for stroke or systemic embolism prevention in AF, and their use is only based on their proven efficacy and safety in VTE.

The use of a DOACs in cancer patients with AF has not been evaluated in a dedicated RCT. However, secondary analyses of phase III DOAC trials using direct factor Xa inhibitors [72,73,74] suggest that DOACs have better safety profiles and are not inferior to VKA on comparison [62]. DOACs should be considered for stroke prevention (excluding patients with moderate-to-severe mitral stenosis or mechanical heart valves) in patients without a high bleeding risk, significant DDIs, or severe renal disease (Class IIa; Level of Recommendation: B). Left atrial appendage (LAA) occluder devices are used in selected cancer patients (Class IIb; Level of Recommendation: C) [62].

Long-term anticoagulant therapy is recommended in adult patients with CHA2DS2-VASc score ≥2 in men or ≥3 in women and must be considered also when the score is 1 in men and 2 in women (Class I; Level of Recommendation: C) [62].

## 4. Anti-Inflammatory Effects of DOACs

### 4.1. Rivaroxaban

The potential anti-inflammatory properties of DOACs have so far been assessed mainly with rivaroxaban (Table 1) [75,76,77,78,79,80,81,82,83,84,85].

Four cellular models have been used to provide insights into the pleiotropic actions of rivaroxaban [75,76,77,78]. Proinflammatory cytokines, chemokines, and adhesion molecules were negatively modulated after rivaroxaban exposition. In human atrial cells, rivaroxaban decreased expression of PARs, IL-8, and ICAM-1 [75]. Besides a reduced PARs expression, rivaroxaban also inhibited PAR-signaling in HUVECs, abolishing transcription of chemokines, MCP-1, and tissue factor (TF) [78]. The reduced production of chemiotactic factors confirmed previous findings from Ishibashi et al. [76,77].

Five in vivo studies were conducted in different mouse models [79,80,81,82,83]. The anti-inflammatory effects were consistent across the experiments. Vascular inflammation was reduced regardless of the etiology [79,83]. Indeed, rivaroxaban prevented recruitment of inflammatory cells limiting MCP-1 and IL-6 generation. [79,83] Moreover, key inflammatory molecules (TNF-a and IL-1) or upstream transcription factors such as NF-Kb were successfully tackled in the remaining investigations [80,81,82]. Finally, two observational studies [84,86] and a post-hoc analysis of the X-VeRT RCT [85] showed that rivaroxaban reduced the inflammatory biomarkers among patients with both AF [84,86] and coronary and periphery artery disease [86].

**Table 1:** Anti-inflammatory effects of rivaroxaban.

Author	Protocol	Target	Disease Model	Results
Bukowska [75]	Pre-clinical in vitro	Human atrial tissue cells	Atrial tissue cultivated with Fxa and stimulated with 4 Hz pacing	↓ PARs ↓ ICAM-1 ↓ IL-8
Ishibashi [76]	Pre-clinical in vitro	HUVECs	Citrated human plasma-induced ROS generation and adhesion molecules expression	↓ MCP-1 ↓ ICAM-1
Ishibashi [77]	Pre-clinical in vitro	Human proximal tubular cells	AGE exposition	↓ MCP-1
Ellinghaus [78]	Pre-clinical in vitro	HUVECs	Thrombin exposition and recalcified plasma incubation	↓ VCAM-1, ICAM-1, MCP-1 ↓ IL-8 ↓ CXCL1, CXCL2 ↓ TF
Zhou [79]	Pre-clinical in vivo	ApoE-deficient mice	Atherosclerosis of innominate artery	↓ MCP-1 ↓ IL-6, TNF-α
Hara [80]	Pre-clinical in vivo	C57BL/6 mice	Atherosclerosis of aortic arch	↓ IL-1β, TNF-α
Imano [81]	Pre-clinical in vivo	Male C57BL/6 J mice	Intermittent hypoxia	↓ NF-kB
Daci [82]	Pre-clinical in vivo	Wistar rats	LPS-induced acute inflammation	↓ MCP-1 ↓ IL-6
Abdelzaher [83]	Pre-clinical in vivo	Adult male Wistar rats	CUMS-induced depression	↓ NF-kB
Martins [84]	Observational study	127 AF patients	-	↓ IL-2, IL-4, IL-10, TNF-α
Kirchhof [85]	Post-hoc analysis of RCT	918 AF patients	-	↓ IL-6
Russo [87]	Observational study	44 CAD/PAD patients	-	↓ IL-6 ↓ fibrinogen

PARs: protease-activated receptors; ICAM-1: intercellular adhesion molecule-1; IL-8: Interleukin-8; HUVECs: human umbilical vein endothelial cells; MCP-1: monocyte chemoattractant protein-1; VCAM-1: vascular adhesion molecule-1, CXCL: C-X-C motif chemokine ligand; TF: tissue factor; ApoE: Apolipoprotein E; IL: Interleukin; TNFa: tumor necrosis factor alpha; NF-kB: nuclear transcription factor kB; LPS: lipopolysaccharide; CUMS: chronic unpredicted mild stress; AF: atrial fibrillation; RCT: randomized controlled trial, CAD: coronary artery disease; PAD: peripheral artery disease.

### 4.2. Apixaban

The current literature does not offer many studies testing the anti-inflammatory effects of apixaban (Table 2). Two in vitro studies demonstrated that cell culture incubation with apixaban limited expression of MCP-1 and adhesion molecules. [88,89] Additionally, in the model replicating the uremic-induced endothelial dysfunction, apixaban mitigated platelet adhesion to extracellular matrix and reactive oxygen species production. [89] Despite little preclinical evidence, a recent longitudinal study on 44 patients with acute cardioembolic ischemic stroke showed lower levels of IL-6 among those in the apixaban group [90].

### 4.3. Edoxaban

Only a few studies assessed the anti-inflammatory properties of edoxaban [91,92]. Although some investigations reported pleiotropic effects of edoxaban such as ROS reduction, anti-angiogenesis, and a decrease in the atherosclerotic plaque burden [93], we chose to report only studies focused on crucial inflammatory molecules (Table 3). In Fang et al. [91], randomized male wild-type mice were given edoxaban or a vehicle diet. Mice in the treatment group exhibited lower inflammatory biomarker levels. Additionally, HK2 cells exposed to edoxaban had suppressed NF-kB expression. Candido et al. [92] examined hospitalized patients with DVT. Participants initially received LWMH according to the guidelines and then were treated with either edoxaban or dabigatran. The 20 patients with DVT demonstrated a lower IL-6 expression when compared to the controls.

### 4.4. Dabigatran

A variety of preclinical studies highlighted the anti-inflammatory effects of dabigatran (Table 4). Hypoxia-exposed rat brain cells subjected to dabigatran showed a blunted inflammatory response with reduced expression of both IL-6 and MCP-1. A simultaneous analysis of brain sections from a transgenic mouse model of neurodegenerative disease yielded similar results [94]. Reduction of TNF-a and IL-1 was consistent across different studies evaluating the effectiveness of dabigatran in models of liver fibrosis, [95] tubulointerstitial fibrosis [96], and acute myocardial infarction [97]. Moreover, dabigatran-ameliorated diabetes-induced endothelial dysfunction fixed the expression of adhesion molecules [98]. Finally, the RIVAL-AF study compared anti-inflammatory effects of dabigatran and rivaroxaban in Japanese patients with non-valvular AF [99]. Dabigatran-treated patients had reduced levels of IL-6. Interestingly, although evaluating DOACs’ anti-inflammatory properties in different clinical scenarios, all DOAC clinical studies reported in this review showed a decreased expression of IL-6, suggesting a potential class effect.

## 5. Anticancer Effects of DOACs

### 5.1. Dabigatran

Most of the anticancer effects have been observed with dabigatran, paradoxically the only DOAC not yet recommended by guidelines in cancer patients (Table 5). A previous study clarified that several classes of proteases, including thrombin, work by directing cell activities through PARs [100]. Tumor initiation and growth, angiogenesis and metastatic spread are all influenced by thrombin.

An in vitro study, by DeFeo et al. [101], firstly described the anticancer properties of DOACs, showing that dabigatran decreased the spread of MDA-MB-231 breast cancer cells; this finding was also confirmed in vivo. Moreover, the authors explored potential antimetastatic effects of dabigatran, reporting a reduction in liver micrometastases when 4T1-cell-injected BALB/c mice were treated with dabigatran.

Alexander et al. evaluated the synergism of dabigatran either with cyclophosphamide or cisplatin [102,103]. Dabigatran alone considerably decreased circulating TF microparticles, even if the tumor suppression required co-administration with cyclophosphamide [102]. In a model of murine ovarian cancer, the co-treatment of dabigatran and cisplatin successfully reduced tumor growth and ascites [103]. Both studies [102,103] showed a reduction in the levels of proinflammatory cytokines and immunosuppressive cells with an established role in tumor promotion.

The potentiation of anticancer effects of common chemotherapeutic agents by dabigatran was shown not only for alkylating agents but also for antimetabolites. Indeed, in a murine model of pancreatic cancer, Shi et al. [105] found an increased gemcitabine cytotoxicity when dabigatran was co-administered. Finally, Vianello et al. [104] provided further mechanistic insights. Dabigatran prevented upregulation of cyclin D1 and proangiogenic factors in both glioblastoma and breast cancer cells; in addition, levels of p27 were restored.

### 5.2. Apixaban

Three in vitro studies explored the anticancer effects of apixaban [108,109,110] (Table 5). Guasti et al. [108] showed that the addition of apixaban to cancer cell cultures, i.e., epithelial cells of ovarian adenocarcinoma (OVCAR3), breast ductal cancer (MDA-MB 23), and colorectal adenocarcinoma (CaCO-2), significantly reduced proliferation and increased mortality of cancer cells through apoptosis in several cancer cell lines, except for the epithelial cells of prostate adenocarcinoma (LNCaP). No effect was noted on histiocytic lymphoma cells (U937). Conversely, cell vitality was lower for all cell lines, both solid and hematological. However, these effects are related to apixaban doses higher than those used clinically. Unfortunately, high doses of apixaban caused cytotoxic effects in endocervical cancer cells (HeLa) [109]. Featherby et al. [110] demonstrated an apixaban-mediated inhibition of proteolytic activity of FVII-TF complex; this effect prevents PAR-2 activation and subsequent release of TF-microvesicles, limiting proliferation.

### 5.3. Rivaroxaban

Besides conventional anticancer effects, such as the cytotoxic and antiproliferative effects investigated for other DOACs, rivaroxaban has proven antiangiogenic properties [106] and interesting effects on antitumor immunity [107] (Table 5).

In mice injected with T241 fibrosarcoma cells, Graf et al. [107] showed a reduction in tumor growth and metastases, suggesting that rivaroxaban promotes antitumor immunity by enhancing infiltration of dendritic cells and cytotoxic T cells at the tumor site. The combination of rivaroxaban and immunotherapy provided a synergistic effect, further limiting tumor growth compared to monotherapy. In particular, rivaroxaban worked on tumor microenvironment promoting CD103 + F4/80 − CCR7 + DC and GrB + cytotoxic CD8 + T cells. Their expression has been linked with better outcomes in cancer patients [112,113]. Moreover, immune-escape pathways supported by macrophages were blunted, as shown by a marked reduced expression in key markers (Mrc1, CD204).

### 5.4. Edoxaban

Only one study investigated the potential anticancer effects of edoxaban [111] (Table 5). In the first phase, all DOACs were studied in BALB/c mice injected with Colon26 cells. Since edoxaban exerted a greater suppression of tumor growth, it was chosen for further analysis. Plasmatic levels of prothrombotic factors such as TF, PAI-1, and MMP-2 were reduced. Additionally, the transcription of STAT3 and cyclin D1 was lowered, while p53 expression was upregulated.

## 6. Anti-inflammatory and Anticancer Effects of Heparins

Heparins are polysaccharides, belonging to the glycosaminoglycan (GAG) family, with anticoagulant effect due to their ability to bind antithrombin III (ATIII). Following the ATIII linkage, heparins increase the inhibitory effect of ATIII towards thrombin and factor X. Moreover, heparins reduce the prothrombotic state by inducing the cells to release tissue factor pathway inhibitor (TFPI), which exhibits both antimetastatic and antiangiogenic properties. 

LMWHs are derived from chemical/enzymatic depolymerization of unfractionated heparin (UFH) and exhibit their anticoagulant function by activating the antithrombin and inhibiting the activation of factor X. LMWHs show more predictable functions and fewer adverse effects compared to UFH.

Besides the well-known anticoagulation effects, heparins seem to be involved in the regulation of the inflammation process, acting on neutrophil migration, complement activation, and cytokine production. The potential anticancer effects of heparins may be attributed to the reduction of prothrombotic state associated to malignancies. Moreover, a protective effect related to inhibiting of cancer progression by interfering with the metastatic process has been described [114,115,116].

Neoplastic cells present an altered expression of adhesion proteins and highly exceed proliferation rate when compared to normal cells. The augmented expression of selectin ligands may increase the metastatic process. Heparins bind to P-selectin, expressed by the platelets, preventing the interaction between platelets and neoplastic cells and reducing the probability of metastasis.

In a mouse model of colon carcinoma and melanoma, Hottlsler et al. showed a lower rate of metastases after heparin treatment. No effect of heparins was observed in mice deficient in P- and L-selectin, hence demonstrating the selectin-dependent function of heparin. [117] Heparin also reduced melanoma metastases in lungs by binding P-selectin expressed on endothelial cells [118] (Table 6). Tinzaparin seems to be the most effective in reducing metastatic spreading by the inhibition of P-selectin.

Growth factors are involved in neoplastic cell proliferation and angiogenesis. GAG mediates the signaling of growth factors and prevents their degradation. Heparins act as a bridge between growth factors and their receptors since they interact with both of them. Exogenous heparins, competing with GAG in the linkage to growth factors, may inhibit fibrosis and angiogenesis. However, only heparins with less than eight sugar units inhibit the VEGF/VEGF receptor interaction; in contrast, huge molecules seem to potentiate the tissue factor signaling, leading to a fast progression of cancer [116,117,118]. Other antiangiogenic potential effects of heparins may be related to the downregulation of factor twist and microRNA-10b (miR-10b), which are involved in cell migration and angiogenesis [122]. They also interfere with heparanase, an endoglycosidase overexpressed in cancer that cleaves heparan sulfate (HS), contributing to extracellular matrix remodeling and the spread of cancer cells [114,115,116,117,118,122,123].

Moreover, heparins showed a pro-apoptotic effect in peripheral blood leukocytes. In nasopharyngeal carcinoma cell line CNE2, heparin increased c-myc expression and bax/bcl2 ratio, both proteins involved in apoptosis regulation. [121]

In two in vitro studies, heparins increased sensitivity to chemotherapy and decreased the chemoresistance [119,120]. Enoxaparin plus gefitinib showed a more significant effect in reducing lung cancer volume and cell migration than gefitinib arm alone; these findings could be justified by a suppression of DOCK1 and AKT pathway, involved in cell migration and proliferation [119]. Moreover, heparins restored responsiveness to cisplatin in cisplatin-resistant ovarian cancer cells [120].

In a meta-analysis including nine studies with 2185 patients with lung cancer, the use of anticoagulant (VKA and subcutaneous heparin) showed a survival benefit and also prolonged life expectancy, especially for those with small-cell lung cancer (SCLC), even if anticoagulation was not indicated [124]. In an RCT by Ek et al. including 380 patients, the addition of enoxaparin at a supraprophylactic dose (1 mg/kg) to the standard treatment did not improve the overall survival, despite resulting in a significant reduction in VTE incidence. These results provides strong support against a more general use of LMWH as a tumor-inhibiting agent and underlines the need for risk biomarkers to guide clinicians in tailoring individualized LMWH treatment [86].

## 7. Clinical Implications

Several experimental in vitro and in vivo studies demonstrated the anti-inflammatory and anticancer effects of DOACs and LWMHs; however, to date, only the anti-inflammatory properties have been shown in studies involving humans. All currently approved DOACs, except dabigatran, are recommended as alternatives to LWMHs in the management of cancer-related VTE. Before starting anticoagulation therapy, a careful cardio-oncologic evaluation is required, as patients with gastrointestinal and genitourinary malignancy showed an increased risk of major bleeding when they were on DOACs therapy. A patient-centered approach based on the clinical characteristics and biomarker evaluation may help clinicians to choose the optimal anticoagulation strategy in patients with malignancy. In the era of precision medicine, the pleiotropic effects of anticoagulants are of significant interest and should be considered alongside drug–drug interactions and benefit/risk balance of drugs. Unfortunately, clinical data for direct anticancer effects are still lacking [125,126]. The “Thrombin Inhibition Preoperatively (TIP) in early breast cancer” is a clinical trial aiming to determine whether 14 days of a preoperative oral factor Xa inhibitor (rivaroxaban) in estrogen-receptor-negative early breast cancer patients results in inhibition of tumor proliferation determined by a reduction in tumor Ki67 from baseline (pre-treatment) to 14 days post treatment start (at the time of surgical excision).

## 8. Conclusions

Inflammation is a common pathway of thrombotic diseases and cancer. Several studies, both in vitro and in vivo, showed “pleiotropic” anti-inflammatory and anticancer effects of DOACs and LMWHs in cellular and animal models. However, to date, only the anti-inflammatory properties have been shown in clinical studies. Further studies are necessary to assess if this experimental evidence may have an impact on patients’ prognosis and overall survival. The use of DOACs as “adjuvant” treatment to chemotherapy, regardless of the patient’s thromboembolic risk, is an intriguing scenario that should be explored with further randomized clinical trials.

## Figures and Tables

**Table 2:** Anti-inflammatory effects of apixaban.

Author	Protocol	Target	Disease Model	Results
Ishibashi [88]	Pre-clinical in vitro	Human kidney mesangial cells	Citrated human plasma-induced ROS generation and adhesion molecule expression	↓ MCP-1, ICAM-1
Nakase [90]	OPS	44 patients with ICS	-	↓ IL-6, hs CRP
Torramade-Moix [89]	Pre-clinical in vitro	HUVECs and HMEC-1	Exposition to serum from uremic patients undergoing peritoneal dialysis	↓ ICAM-1, VCAM-1

ROS: reactive oxygen species; MCP-1: monocyte chemoattractant protein-1; ICAM-1: intercellular adhesion molecule-1; OPS: observational prospective study; IL-6: Interleukin-6; hsCRP: high-sensitivity C reactive protein; ICS: ischemic cardioembolic stroke; HUVECs: human umbilical vein endothelial cells; HMEC-1: human microvascular endothelial cells; VCAM-1: vascular adhesion molecule-1.

**Table 3:** Anti-inflammatory effects of edoxaban.

Author	Protocol	Target	Disease Model	Results
Fang [91]	Pre-clinical in vitro	Male wild-type mice and HK-2 cells	5/6 nephrectomy surgery	↓ TNFa ↓ MCP-1
Candido [92]	CC study	42 DVT patients	-	↓ IL-6

HK-2: human kidney 2 cells; TNFa: tumor necrosis factor alpha; MCP-1: monocyte chemoattractant protein-1; CC: case control; DVT: deep-vein thrombosis; IL: Interleukin.

**Table 4:** Anti-inflammatory effects of dabigatran.

Author	Protocol	Target	Disease Model	Results
Tripathy [94]	Pre-clinical in vitro	Rat brain endothelial cell cultures	Hypoxia exposition	↓ MCP-1
Song [97]	Pre-clinical in vitro	Male New Zealand white rabbits	Coronary-occlusion-induced acute myocardial infarction	↓ TNFa, IL-1 ↓ NF-kB
Mahmoud [95]	Pre-clinical in vitro	Adult male albino rats	CCl4-induced liver fibrosis	↓ TNFa, IL-1
Saifi [96]	Pre-clinical in vitro	Male Swiss albino mice	UUO-induced renal fibrosis	↓ TNFa, IL-1
Rahadian [98]	Pre-clinical in vivo	STZ-induced diabetic C57BL/6 J mice	STZ-induced diabetes	↓ MCP-1, ICAM-1
Kikuchi [99]	RCT	117 AF patients	-	↓ IL-6, IL-18

MCP-1: monocyte chemoattractant protein-1; TNFa: tumor necrosis factor alpha; CCl4: carbon tetrachloride; UUO: unilateral ureteral obstruction; STZ: streptozotocin; IL-1: Interleukin-1; NF-kB: nuclear transcription factor kB; ICAM-1: intercellular adhesion molecule-1; IL-6: Interleukin-6; IL-8: Interleukin-8; AF: atrial fibrillation; RCT: randomized controlled trial.

**Table 5:** Anticancer effects of DOACs.

Author	Protocol	DOAC	Target	Disease Model	Results
DeFeo [101]	Pre-clinical in vitro and in vivo	Dabigatran	MDA-MB-231 cells, 4T1 cells and BALB/c mice	Breast cancer	↓ Cell migration both in vitro and in vivo ↓ Circulating 4T1 cells ↓ Tumor dimension ↓ Liver micrometastases
Alexander [102]	Pre-clinical in vitro and in vivo	Dabigatran	4T1 cells and BALB/c mice	Breast cancer	↓ Tumor dimension ↓ Lung metastases ↓ TGFβ ↓ Arginase + Gr-1 + CD11b+ MDSCs ↓ splenomegaly ↓ Circulating TF microparticles ↓ Activated platelets
Alexander [103]	Pre-clinical in vitro and in vivo	Dabigatran	ID8-luc mouse ovarian carcinoma cells andC57/Bl6 mice	Ovarian cancer	↓ Tumor growth ↓ Ascites ↓ Circulating activated platelets ↓ Circulating TF microparticles ↓ Gr1+/CD11b+ MDSCs ↓ CD11b+/CD11c+ DCs ↓ TGF-β, VEGF, IL-6, IL-10, and MCP-1↑ IFN-g
Vianello [104]	Pre-clinical in vitro	Dabigatran	MDA-MB-231 and U87-MG	Breast cancer, glioblastoma	↓ Thrombin-induced: —Tumor proliferation —p27 downregulation —Cyclin D1 upregulation—Proangiogenetic protein expression —Vascular tube formation —Cell migration
Shi [105]	Pre-clinical in vitro and in vivo	Dabigatran	Panc02 cells and C57Bl/6 mice	Pancreatic cancer	↓ Tumor growth ↓ Cell dissemination
Yavuz [106]	Pre-clinical in vivo	Rivaroxaban	Ross 308 strain fertilized hen eggs	CAM	↓ Angiogenesis
Graf [107]	Pre-clinical in vitro and in vivo	Rivaroxaban	Melanoma B16F10 cells, fibro-sarcoma T241, PyMT tumor cells, and mutant mice	Breast cancer, melanoma, fibrosarcoma	↓ Tumor growth ↓ Metastases ↓ TAMs expression of Mrc1 CD204 ↓ FoxP3 + CD4+ T cells in TME ↑ Tumor-killing granzyme B+ CD8+ T cells ↑ CD169+ macrophages and CD8+ DCs ↑ Antitumor immunity
Guasti [108]	Pre-clinical in vitro	Apixaban	OVCAR3, MDA-MB-231, CaCO-2, LNCaP, U937 cells	Ovarian cancer, breast cancer, colon cancer, prostate cancer, histiocytic lymphoma	↑ p16 ↑ Cell mortality (OVCAR3, MDA-MB-231, CaCO-2, U937 cells) ↓ Cell migration (OVCAR3, CaCO-2) ↓ Cell Proliferation (OVCAR3, CaCO-2, LNCaP)
Kubat [109]	Pre-clinical in vitro	Apixaban	HeLa cells	Cervical cancer	↓ Cell viability
Featherby [110]	Pre-clinical in vitro	Apixaban	MDA-MB-231 and AsPC-1 cells	Breast cancer, pancreatic cancer	↓ Release of TF-microvesicles from both fXa-activated cells and non-activated cells ↓ Cell proliferation of both fXa-activated cells and non-activated cells
Hiramoto [111]	Pre-clinical in vitro and in vivo	Edoxaban	Colon26 cells and BALB/c mice	Colon cancer	↓ Tumor growth ↓ TF, PAI-1, IL-6, and MMP-2 ↓ PAR2, STAT3, cyclin D1, and Ki67

MDSCs: myeloid-derived suppresser cells; DCs: dendritic cells; TAM: tumor-associated macrophages; TME: tumor microenvironment; CAM: chick chorioallantoic membrane; TF: tissue factor; PAI-1: plasminogen activator inhibitor 1; MMP-2: metalloproteinase 2; PAR2: protease-activated receptor 2; STAT3: signal transducer and activator of transcription 3.

**Table 6:** Anticancer effects of heparins.

Author	Protocol	Target	Disease Model	Results
Hostettler [117]	Pre-clinical in vitro and in vivo	MC-38, B16-BL6 cells and C57BL/6 J mice	Colon carcinoma/melanoma	↓ Metastases
Ludwig [118]	Pre-clinical in vitro and in vivo	B16F10 cells and C57BL/6 J mice	Melanoma	↓ Metastases
Pan [119]	Pre-clinical in vitro and in vivo	NSCLC-A549, H1975 cells and BALB/c mice	Non-small-cell lung cancer	↓ Tumor volume, cell migration
Pfankuchen [120]	Pre-clinical in vitro	A2780 cells	Ovarian cancer	↑ Cisplatin sensitivity
Li [121]	Pre-clinical in vitro	CNE2 cells	Nasopharyngeal carcinoma	↑ DNA fragmentation, apoptosis, bax/bcl2 ratio
Shen [122]	Pre-clinical in vitro	HMEC-1 cells	-	↓ MiR-10b expression ↓ Cell migration, tube formation, and angiogenesis

MC-38: murine cell line of colorectal cancer; B16-BL6: murine cell line of melanoma; NSCLC-A549: non-small-cell lung cancer cisplatin-resistant cell lines; H1975: human cell line of lung adenocarcinoma; A2780: human cell line of ovarian cancer; CNE2: human cell line of nasopharyngeal carcinoma; HMEC-1: human microvascular endothelial cells.

## Data Availability

Not applicable.
